# An Assessment of the Influence of the Industry Distribution Chain on the Oxygen Levels in Commercial Modified Atmosphere Packaged Cheddar Cheese Using Non-Destructive Oxygen Sensor Technology

**DOI:** 10.3390/s16060916

**Published:** 2016-06-20

**Authors:** Karen A.M. O’ Callaghan, Dmitri B. Papkovsky, Joseph P. Kerry

**Affiliations:** 1 Food Packaging Group, School of Food and Nutritional Sciences, University College Cork, Cork T12 YN60, Ireland; k.a.ocallaghan@umail.ucc.ie; 2 Biophysics and Bioanalysis Group, School of Biochemistry and Cell Biology, University College Cork, Cork T12 YN60, Ireland; d.papkovsky@ucc.ie

**Keywords:** sensor, oxygen, packaging, distribution, cheese, industry

## Abstract

The establishment and control of oxygen levels in packs of oxygen-sensitive food products such as cheese is imperative in order to maintain product quality over a determined shelf life. Oxygen sensors quantify oxygen concentrations within packaging using a reversible optical measurement process, and this non-destructive nature ensures the entire supply chain can be monitored and can assist in pinpointing negative issues pertaining to product packaging. This study was carried out in a commercial cheese packaging plant and involved the insertion of 768 sensors into 384 flow-wrapped cheese packs (two sensors per pack) that were flushed with 100% carbon dioxide prior to sealing. The cheese blocks were randomly assigned to two different storage groups to assess the effects of package quality, packaging process efficiency, and handling and distribution on package containment. Results demonstrated that oxygen levels increased in both experimental groups examined over the 30-day assessment period. The group subjected to a simulated industrial distribution route and handling procedures of commercial retailed cheese exhibited the highest level of oxygen detected on every day examined and experienced the highest rate of package failure. The study concluded that fluctuating storage conditions, product movement associated with distribution activities, and the possible presence of cheese-derived contaminants such as calcium lactate crystals were chief contributors to package failure.

## 1. Introduction

Oxygen is directly or indirectly linked to the major incidences of spoilage associated with hard cheeses. Modified atmosphere packaging (MAP) utilizes gas mixes deficient in oxygen to extend the shelf life of hard cheeses by affording protection against oxidation and the proliferation of undesirable spoilage microorganisms. Consequently, hard and semi-hard cheeses are commonly surrounded by laminate combinations of polyamide and polyethylene [1], and packed in 100% carbon dioxide or mixtures of carbon dioxide and nitrogen using horizontal form-fill-seal pouch pack equipment [2]. The use of 100% carbon dioxide is favored by many cheese packers, as the employment of this MAP approach typically encourages inhibition of microbial growth, but, more specifically, produces a cheese pack with the physical appearance of a vacuum package. This occurs because carbon dioxide has a high solubility in high-moisture/high-fat foods at low storage temperatures, such as cheese, and, when applied in excess, can result in a fully intentional package collapse [3], which is technically described as “snug-back” or “snug-down.” This gas absorption equilibrium process (between the headspace and the product) is known to be relatively fast and is obtained within the first few days after packaging [4].

However, if the package, packaging process, storage, or distribution conditions fail to contain hard cheese products properly, then the benefits imposed by modifying the atmosphere will be ineffective, and “snug-back” or “snug-down” will not occur, thereby negatively affecting the visual appearance of the final retail pack. Additionally, when such primary packaged cheese products have been manufactured for export, the primary packaging must contend with more challenging stresses from the point of collation, handling, and distribution purposes through extended distances and environmental conditions presented as the primary packs move through the cold-chain distribution system employed in the market placement of such products. Therefore, monitoring the level of oxygen within the package can give critical information on the status of cheese quality and shelf life, and assist in the pinpointing of negative containment issues as they arise from the point of product manufacture to the point of retail purchase.

Traditional methods used to determine the presence of oxygen within packaging headspaces are usually of a destructive nature. The tests are irreversible; products must be analyzed in a batch-wise manner (continuous assessment cannot be achieved) and are unsuitable for identifying leaks. Non-destructive optical oxygen sensors quantify oxygen concentrations by measuring the luminescence quenching effect of oxygen [5]. Sensors (solid supports containing an oxygen sensitive dye) are monitored using a portable detector, which emits a light source causing an electronic excitation of the dye. The long-lived emission of the phosphorescent dye molecules allows for the deactivation of their excited state through the collisional interaction with oxygen molecules, which decreases the emission intensity (I) and lifetime (τ) of the dye in a concentration-dependent manner [6]. The detector simultaneously measures the changes in phase shift of the phosphorescence (I, τ), allowing oxygen concentration to be calculated by the instrument software using a predetermined calibration function [7]. Further details on the operation and performance of these sensors have been described in [6,8,9]. The fundamental advantage of the employment of the sensors is that the measurement is a non-destructive reversible process, and the entire supply chain can therefore be monitored, and control points can be identified. 

Previously, oxygen levels using sensor technology in cheese packages were evaluated in both [10,11] (*n* = 67 and *n* = 40, respectively). However, both studies had small sample sizes, and neither determined the influence of distribution on package function. This investigation concentrates on a larger sample size (384 cheese blocks) in an industrial setting and assesses the influence of distribution on packaging containment by measuring oxygen levels in the cheese packages using non-destructive oxygen sensor technology. 

## 2. Experimental Section

### 2.1. Oxygen Sensor Manufacture and Calibration

Oxygen sensors based on a phosphorescent oxygen-sensitive dye (Platinum octaethyl porphyrin-ketone—Pt-OEPK) spotted on microporous support [12] were supplied by Luxcel Biosciences (Cork, Ireland). Pt-OEPK-based sensors are highly sensitive to oxygen (~0.02%), are photochemically stable, display good compatibility with semiconductor optoelectronics (e.g., excitation by light-emitting diodes), possess excellent storage and operational stability, and are suitable for use with a wide range of products and applications [9,13]. Each sensor (6-mm disc) was attached to the center of an adhesive sticker (Avery Dennison, CA, USA). The sensor sticker was reapplied to the backing tape for mobility purposes and to maintain adhesion prior to package insertion. Sensors were calibrated using Optech^®^ Platinum handheld reader (Mocon Inc., Minneapolis, MN, USA) with two standard gas mixtures (0% oxygen and air), and the resulting calibration was stored in Optech^®^ software and applied to the whole batch of sensors. 

### 2.2. Application of Sensors 

Sensors (*n* = 768) were incorporated into cheese packs during the normal industrial packaging process at a local cheese packaging plant. The production line was slowed down to allow two sensors to be placed directly on the cheese, at each end of the cheese block. Packages were made of orientated polyamide (15 µm) and polyethylene (50 µm), a laminate with low oxygen permeability (<30 cm^3^/m^2^/24 h/atm). The cheese was packaged under standard packaging conditions—horizontal form, fill and seal, and flushed with 100% carbon dioxide prior to sealing. An online gas analyzer (Dansensor, Ringsted, Denmark) read the initial oxygen levels to confirm that oxygen was not present in all packs. Packs that read an oxygen level greater than 0.5% were removed from the line and repackaged. 

### 2.3. Experimental Storage Treatments 

A total of 384 cheese blocks (~1 kg) were manufactured to contain the sensor technology in this study. Blocks were randomly designated to one of two experimental storage treatments, namely, A and B, with each cheese block assigned an identification number. Treatment groups A and B both contained 12 independent secondary corrugated paperboard boxes (160WTK/160SC/170T3 R Flute), each of which held 16 primary packaged cheese blocks stacked as per the usual structure employed for distribution formats (16 cheese blocks × 12 boxes = 192 cheese blocks per treatment). The two experimental storage groupings were as follows.

Group A—This group was stored under refrigerated conditions (4 °C) at the cheese packaging plant and subjected to minimal movement. Package fault could be due to a permeation issue or due to insufficient film thickness, or it could be some aspect of the packaging process (e.g., alignment, gases applied, or sealing) that is not executing its purpose. Therefore, storage Group A examined the performance of the packaging process and package function. 

Group B—Boxes were palletized and transported offsite via a commercial refrigerated (4 °C) truck and kept at an external storage facility overnight under refrigerated conditions. Palletized product was off-loaded and re-loaded using a forklift at the storage facility to further simulate commercial handling stresses. This group was then returned back to the original cheese packaging plant for assessment. The round-trip distance experienced by cheese group B was approximately 100 km. Storage group B determined whether the packaging function becomes compromised through the conditions experienced during simulated transport and handling procedures, which are experienced during the industrial distribution of retailed cheese.

### 2.4. Measurement of Oxygen Sensors

Oxygen measurements were recorded using an Optech^®^ Platinum handheld reader (Mocon Inc.), which was connected to a laptop via a serial port. The Optech^®^ detector acquires a measurement (e.g., changes in phase shift) from an oxygen sensor within the package by placing the tip of the detector probe over the sensor. The readings were stored in Optech^®^ software and presented in ppm, which was then converted to % oxygen. 

Cheese packs were measured periodically for oxygen levels over a 30-day storage period. Both sensors in each pack were read and an average oxygen reading calculated. Table 1 details the measurement schedule for the trial. On Day 0 (12 h post-packaging), Boxes 1–11 in both storage groups were measured. Group A was monitored more frequently at the beginning of the trial, as any pack containment issues relating to packaging or processing were deemed most likely to occur earlier during the storage period. Box 12 in both groups was deemed a control box and was served to determine whether a lack of routine handling actually reduced the number of containment packaging faults experienced when compared with the more frequently handled packs in Boxes 1 to 11. This box was measured only on Day 30, which signified the end of the storage trial.

### 2.5. Definition of Cheese Pack Failure

A cheese package was deemed to have failed if pack containment was lost, as indicated by an oxygen content that was greater than 1%. Although complete elimination of oxygen is desired and processing procedures in the factory are implemented to remove all oxygen from packages, low levels of oxygen can become trapped within a package for various reasons during the packaging process, and this oxygen is termed residual oxygen. 

### 2.6. Pack Integrity Testing

Package integrity was performed at the conclusion of the 30-day trial period on all cheese packages that demonstrated failure (oxygen reading >1%). Integrity was assessed in two ways and these are described as follows:

- Leak Detection System (PFM, Leeds, UK)

The package was placed into the testing chamber and a vacuum pulled for 30 s at 0.6 bar. If bubbles were not present on testing, the cheese package was deemed to have “passed”. However, if a constant stream of bubbles emerged from the package, then the pack was considered to have failed due to the presence of a leak, and the package was further inspected.

- Submergence Test

The leak detection test was augmented by carrying out the submergence test, which involved the physical submergence of the packed cheese by an operator into a tank of water. This non-pressurized method allows the operator to manipulate the pack underwater. All pack seals were examined and small leaks inspected. Some light pressure was applied to certain pack areas when required in order to ensure that leaks were in fact present. If a leak was detected, the area of the leak was encircled using a permanent marker for further examination.

### 2.7. Headspace Analysis

A commercial gas headspace analyzer CHECK Checkmate^®^ 990 (Dansensor) was used to establish progress within the package of the initial carbon dioxide applied during the packaging process. It was also used to confirm an oxygen level correlation with the non-destructive oxygen sensor measurements taken. This method was used only after Day 30 of storage, as it is a destructive evaluation, and pack integrity becomes compromised post-assessment. The procedure involves applying a neoprene plastic pad to the package and piercing the package through the pad with the needle, ensuring the needle is exposed to the headspace without touching the cheese contained. The needle extracts a sample of gas from the headspace, which is analyzed for both oxygen and carbon dioxide levels. 

### 2.8. Leak Visualisation-Microscopy

A light and fluorescence microscope (Olympus BX61—Mason Technology, Dublin, Ireland) was used to visualize and magnify any leaks detected in the packaging. The packaging materials surface was observed using reflected light. 

### 2.9. Statistical Analysis

The mean oxygen content (%) and standard deviation was calculated for each group over the 30-day storage period. This experimental data was also analyzed on SPSS Statistics 20 (IBM, Armonk, NY, USA). A Paired-Samples T-Test was performed to determine statistical significance between storage groups on each measurement day during the trial, and the level of significance was set at *P* ≤ 0.05. Additionally, a correlation coefficient (R) was calculated (Microsoft Office Excel 2007) to determine the strength of correlation between non-destructive and destructive oxygen sensing techniques.

## 3. Results and Discussion

### 3.1. Measurement of Oxygen Sensors

**Figure 1:**
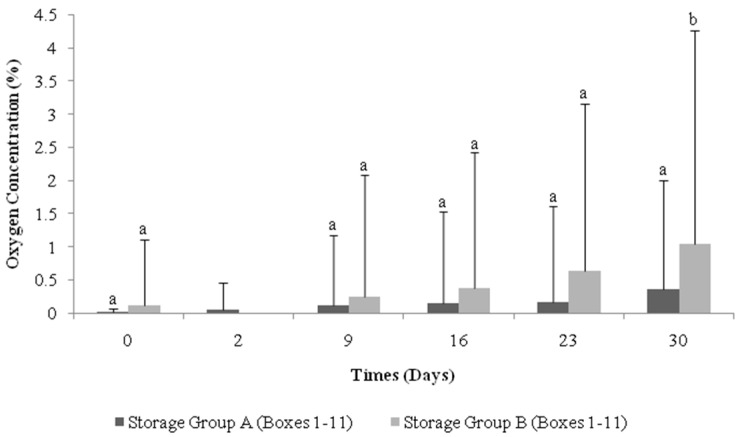
Profile of the mean oxygen content of each storage treatment Group (**A**—not distributed and **B**—distributed) assessed over 30 days. A significant difference (*P* ≤ 0.05) between storage groups on each day is indicated using lowercase lettering (**a**,**b**).

Experimental storage Groups A and B were monitored over a 30-day period and Figure 1 shows the mean oxygen content (%) of each group over this timeline. The oxygen level present in the cheese packs increased over time in both experimental treatment groups, with standard deviation bars demonstrating that variation occurred as a result of the presence of failed packs. In Group A, the mean oxygen level for Day 0 (0.02%) increased to 0.37% by Day 30. Group B cheese packs had an initial oxygen level of 0.12%, and this increased to a mean oxygen content of 1.05%. Our results are in agreement with those in [11], the authors of which observed an increase in oxygen within packs over a 148-h trial period and attributed this elevation to poor packaging procedures. However, both treatment groups in this study were packaged identically, yet Group B cheese packs had a higher oxygen content compared to Group A packs on every day examined throughout the storage period and were found to be statistically different from Group A on Day 30 (*P* ≤ 0.05) (Figure 1), clearly indicating that distribution factors facilitate an increase in oxygen levels within cheese packs. 

### 3.2. Cheese Package Failure

The range-values for final oxygen levels within each treatment group are shown in Table 2, along with the overall % cheese pack failure for each experimental treatment. Group A cheese packs had a rate of failure of 3.13%, which indicated that the primary packaging, the packaging process, or both were not providing the full technical functions necessary to properly contain the cheese packs. When compared to storage Group A, Group B cheese packs were found to have a pack failure rate of 7.29%; more than double the rate of pack failure observed for Group A cheese packs. Group B was the only storage group to leave the packaging facility and was therefore subjected to additional handling and transportation stresses, as well as being exposed to temperature fluctuations. Therefore, these distribution forces are the most probable factors in causing an increased level of containment failure. Rough transportation assists in accelerating the inception of oxygen by creating excessive movement that can cause friction between the cheese product and package, between each cheese package, between the packages and the corrugated paperboard box, and between the boxes and the pallet, plus any other tertiary packaging surround. In addition to interference caused by conveyance variations, temperature fluctuations can affect the package in two ways. Firstly, the permeability of packaging films is a function of temperature and permeability increases as temperature increases [3], which can result in containment failure. Secondly, at increased temperature, the solubility of carbon dioxide decrease; therefore, not only does snug-back fail to be achieved (affecting the visual appearance of the final retail pack), but the extent of antimicrobial activity is reduced as inefficient levels of dissolved carbon dioxide are absorbed by the cheese, thereby failing to provide the full antimicrobial capacity of the product [14]. Additionally, it was hypothesized that the handling of the blocks during the measurement procedure could have contributed to the formation of leaks, thereby leading to more package failures and a false representation of results. Thus, one box in each of the two cheese groupings (Box 12) was used as a control and only measured once on Day 30 of storage. Table 3 shows that failure occurred in this box for both storage groups despite never being opened and never being subjected to routine handling and measurement. This result demonstrates that handling the blocks during measurement minimally affected the rate of pack failure and, furthermore, that oxygen sensors and the associated measurement process can be incorporated into an industrial setting without compromising package function. 

**Figure 2:**
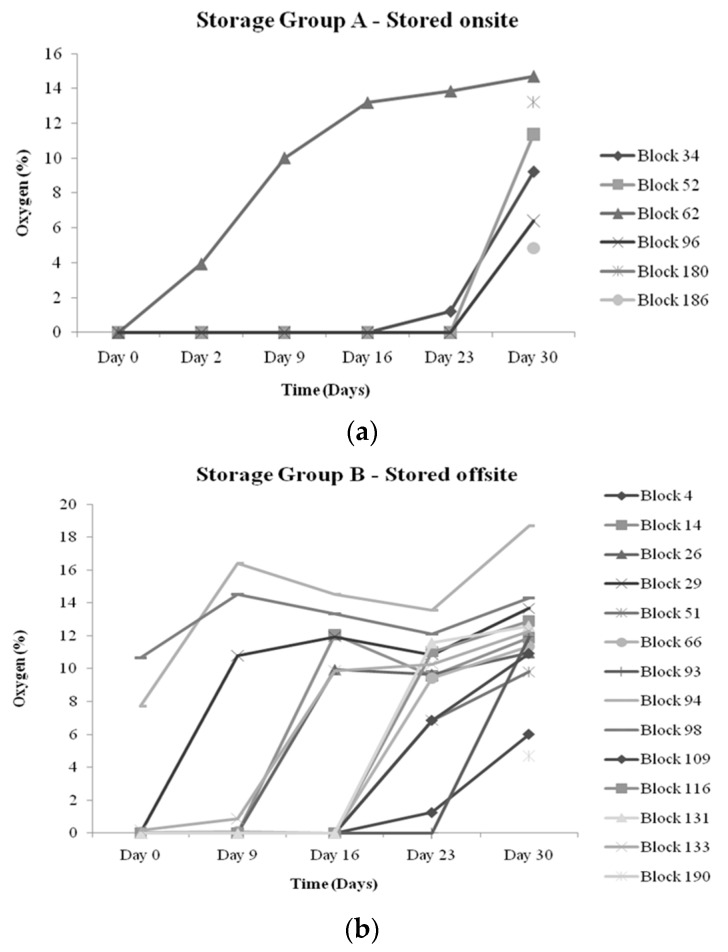
(**a**) and (**b**) Shows the profiles of the progression of oxygen within the packs that diagnosed as failures (oxygen >1%) at any stage over the 30-day measurement period. Some failed blocks presented within Box 12 (control), which was only measured on Day 30. Each point represents the mean oxygen content of each failed pack on each measurement day.

Figure 2a,b display the oxygen profile for the cheese blocks that exhibited failure (>1%) at any measurement point over the 30 days of refrigerated storage. A common feature of both of the oxygen profiles is that, in general, oxygen levels were under specified failure limits on the initial day of testing, with oxygen levels being low at the start of the trial and then experiencing a sudden spike in the oxygen content. This action may be explained by the presence of leaks as opposed to a permeation concern, as gradual increases in oxygen levels are more likely associated with poor permeability properties of the packaging material employed [15]. Damage due to permeability involves changes in the gaseous atmosphere, which occurs slowly, whereas leaks can cause rapid changes in the headspace and can also cause potential moisture and microbial ingression. In [10], the authors demonstrated that packs that presented increased oxygen content and also demonstrated the earliest appearance of mold growth, which is usually not observed until much later. This shows that oxygen sensor measurement is key in predicting a reduced shelf life; therefore, early detection is both informative and advantageous as failed blocks could be repackaged or removed from the supply chain, dependent on the stage of diagnosis.

### 3.3. Package Integrity Testing

The performance of the package and seal function was examined via package integrity tests. The cheese blocks assessed were those that were deemed to have failed (>1% oxygen levels in packs). These included 6 cheese blocks from Group A (Blocks—34, 52, 62, 96, 180, 186) and 14 cheese blocks from Group B (Blocks—4, 14, 26, 29, 51, 66, 93, 94, 98, 109, 116, 131, 133, 190). From a visual assessment, the packs with the highest oxygen readings experienced a “pillow” effect—a billowing or expansion of the package. This illustrates that oxygen had penetrated the inner atmosphere and indicated that the carbon dioxide applied during the packaging process potentially failed to be absorbed by the cheese.

**Figure 3:**
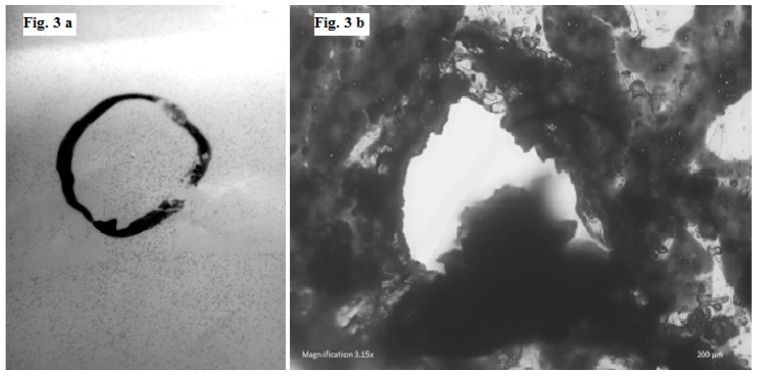
Marked location of the pinhole identified (**a**) and image of pinhole magnified using a light microscope (**b**).

Both integrity tests, which employed the use of water submergence, demonstrated the presence of bubbles emanating from a number of cheese packs. Often the existence of bubbles may indicate a false result and can be due to the occupancy of air entrapped within the seal-folds and at the seal-lips of the pack. Furthermore, if the bubbles present are arising from pack leaks, then detection of the leak can be extremely difficult depending on the pinhole size. Medium- or large-sized pinholes can usually be observed by sight or easily detected via integrity testing. Smaller holes may be more harmful, as they are much harder to identify and can evade detection (bubbles too small or infrequent from cheese packs during water submergence testing); therefore, product deterioration can occur unknowingly. These small pores, microholes, and cracks not only allow the transfer of gas and moisture from the external environment, but also permit microbial penetration into the package, with some bacteria penetrating holes as small as 0.4 µm in diameter [16]. The only visible fault detected was observed as a pinhole in Group B (Block 116). The pinhole position was on the main body on the back of the block; the hole was marked (Figure 3a) and later subjected to microscopy (Figure 3b). This fault verifies that the integrity of the pack was compromised, and any benefit bestowed by packaging in a modified atmosphere was therefore lost. This concern was further explored by examining the headspace atmosphere. 

### 3.4. Headspace Analysis

**Figure 4:**
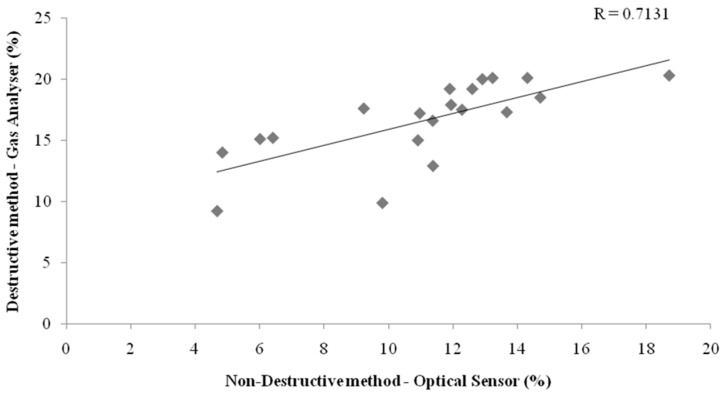
Relationship between oxygen measurement methods (destructive and non-destructive) and their correlation coefficient value (R).

The failed packs were evaluated using headspace analysis post-integrity testing, as this was a destructive method. This test was performed to confirm association between the two methods of oxygen measurement and to ascertain what happens to the carbon dioxide within the package. Oxygen measurements were determined to be positively correlated with a correlation coefficient (R) of 0.7131, which indicates a strong relationship between oxygen sensor levels (non-destructive) and oxygen values obtained from the headspace analyzer (destructive) (Figure 4). Since headspace analysis is of a destructive nature, this measurement was acquired after the 30-day trial had concluded. Therefore, oxygen measurements taken by the gas analyzer had increased levels, suggesting that the correlation may have been greater if the measurements of the two methods had been recorded simultaneously.

**Figure 5:**
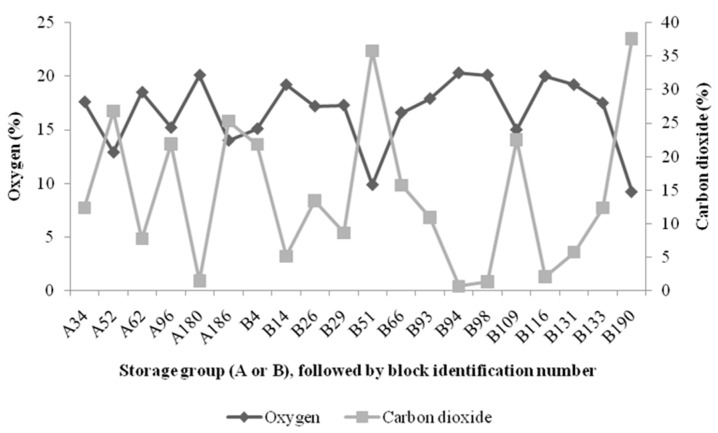
Comparison of carbon dioxide and oxygen readings of failed cheese blocks from storage Groups A and B.

In general, the blocks that registered the lowest oxygen values correspond to the highest carbon dioxide levels (Figure 5). Blocks that were found to be failures exhibited a billowed appearance. The carbon dioxide levels within these packs were lower, which infers the carbon dioxide dissipates from the pack once the leak perpetuates and oxygen continually ingresses. Therefore, the benefit of microbial protection provided by carbon dioxide was lost and the progression of oxygen into the pack would lead to a shortening of shelf life and a deterioration in product quality. 

### 3.5. Microscopy of Packaging Faults

The marked location where the pinhole was discovered through pack integrity testing was visualized using a light and fluorescence microscope (Figure 3b). From physical examination of the pack, it was observed that pinhole formation emanated from the inside of the package, indicating that an entity within the pack was most likely to be the causative agent. Features within the pack that may be responsible include sharp edges presented by the cheese itself from cutting the cheese into blocks, coarseness on the block surface, and the presence of lactate crystals on the exterior of the cheese. The position of our pinhole on the cheese surface suggests that crystals were the most probable cause due to their size, morphology, and potential to create small holes. Crystals in cheese can be both desirable and undesirable. Some cheese manufacturers produce cheeses with a higher lactate crystal content owing to the distinctive sensory profile created by their presence. Calcium lactate crystal formation is influenced by cheese characteristics (manufacturing, shape, maturity, composition, bacteria present), packaging (format, integrity, barrier), storage, and distribution conditions (temperature, light exposure and excessive handling). In particular, it has been noted that gas flushing with carbon dioxide alone can cause faster and greater generation of crystals [17]. Therefore, a modified atmosphere of 100% carbon dioxide and an increase in temperature or movement, can all catalyze crystal production. Storage Group B was specifically subjected to simulated distribution, and the conditions it was exposed to, coupled with their associated promotion of crystal formation, may have contributed to its high rate of failure. Such high levels of failure are unacceptable from a commercial standpoint and could mean significant losses for the cheese industry if not resolved. Approaches could be implemented to reduce crystal development in order to minimize these losses from occurring, however, due to certain manufacturers targeting the increased existence of these crystals, an alternative approach is required. Therefore, there is a need to carefully monitor and control the conditions that occur during packaging, handling, storage, and distribution of hard cheeses in order to avoid excessive product losses owing to pack containment failures. Engagement of both active and intelligent systems, specifically the employment of oxygen sensors, can aid in the regulation of the entire supply chain. 

## 4. Conclusions

As demonstrated in this study, sensors can be utilized successfully at an industrial scale to monitor the extent of abuse from packaging onwards and allow changes to be made to the supply chain following the gathering of information. The mean oxygen content in both experimental storage groupings increased over time. The highest rate of failure occurred in Group B with the source of failure determined to be due to the presence of leaks. The predominant reason for the formation of leaks was found to be due to the distribution conditions experienced and the possible existence of crystals on the cheese surface. Future work should focus on implementing greater control throughout the supply chain to avoid the occurrence of leaks, the incorporation of these non-destructive sensor systems, which are key in recognizing when leakage does occur, and the employment of smart technologies such as antimicrobial packaging, which can provide additional protection should a leak manifest. 

## Figures and Tables

**Table 1:** Sensor measurement schedule *.

Day	Group A	Group B
0	(1–11)	(1–11)
2	(1–11)	x
9	(1–11)	(1–11)
16	(1–11)	(1–11)
23	(1–11)	(1–11)
30	(1–12)	(1–12)

* Group A: Stored onsite and experienced minimal movement; Group B: Subjected to simulated industrial distribution; Boxes measured in brackets, with Box 12 representing as a control.

**Table 2:** Final oxygen ranges for both storage groups (No. of blocks) and rate of failure* (%) on Day 30.

Group	0%–0.5%	0.5%–1%	1%–5%	5%–10%	10%–15%	15%–20%	Total >1%	Failure (%)
A (192 blocks)	184	2	1	2	3	0	6	3.13
B (192 blocks)	173	5	1	2	10	1	14	7.29
Total (384 blocks)	357	7	2	4	13	1	20	5.21

* Failure is defined as levels that exceed 1% oxygen. The oxygen ranges underlined are deemed failures. Group A—Stored onsite and experienced minimal movement. Group B—Subjected to simulated industrial distribution.

**Table 3:** Box 12 (Control) information *.

	Group A	Group B
No. of blocks in box	16	16
No. of failed blocks	2	1

* Control box was never handled and only measured on Day 30. Group A—Stored onsite and experienced minimal movement. Group B—Subjected to simulated industrial distribution.
